# High incidence of classic Kaposi's sarcoma in Mantua, Po Valley, Northern Italy (1989–1998)

**DOI:** 10.1054/bjoc.2001.1912

**Published:** 2001-08

**Authors:** V Ascoli, S Belli, M Benedetti, S Trinca, P Ricci, P Comba

**Affiliations:** Section of Pathology, Department of Experimental Medicine and Pathology, La Sapienza University, Viale Regina Elena, Rome, 324 00161, Italy; Section of Environmental Epidemiology, National Institute of Health (ISS), Viale Regina Elena, Rome, 299 00161, Italy; Local Health Authority (SPSAL/ASL) Provincia di Mantova Via Cesare Battisti, 5 Mantua, 46100, Italy

**Keywords:** Kaposi's sarcoma, human herpesvirus 8, Mantova, Lombardia, Po Valley, Italy

## Abstract

The incidence of classic Kaposi's sarcoma was estimated in the province of Mantua, Po Valley, Northern Italy, yielding age-standardized rates of 2.5/100 000 men and 0.7/100 000 women (1989–98). Elevated rates in the rural zone of Viadana/Sabbioneta (5.0/100 000 men and 2.8/100 000 women) are among the highest so far reported for Italian communities. © 2001 Cancer Research Campaign http://www.bjcancer.com


*British Journal of Cancer* (2001) **85**(3), 379–382
© 2001 Cancer Research Campaign
doi: 10.1054/ bjoc.2001.1912, available online at http://www.idealibrary.com on IDEAL®

http://www.bjcancer.com

Short Communication

### High incidence of classic Kaposi's sarcoma in Mantua, Po Valley, Northern Italy (1989–1998)

**V Ascoli[1], S Belli[2], M Benedetti[2], S Trinca[2], P Ricci[3] and P Comba[2]**

[1]Section of Pathology, Department of Experimental Medicine and Pathology, La Sapienza University, Viale Regina Elena, 324,00161 Rome, Italy, [2]Section of Environmental Epidemiology, National Institute of Health (ISS), Viale Regina Elena, 299,00161 Rome, Italy; [3]Local Health Authority (SPSAL/ASL) Provincia di Mantova, Via Cesare Battisti, 5,Mantua, 46100, Italy

**Summary** The incidence of classic Kaposi's sarcoma was estimated in the province of Mantua, Po Valley, Northern Italy, yielding age-standardized rates of 2.5/100 000 men and 0.7/100 000 women (1989–98). Elevated rates in the rural zone of Viadana/Sabbioneta (5.0/100 000 men and 2.8/100 000 women) are among the highest so far reported for Italian communities. © 2001 Cancer Research Campaign http://www.bjcancer.com

**Keywords**: Kaposi's sarcoma; human herpesvirus 8; Mantova; Lombardia; Po Valley; Italy

The classic variant of Kaposi's sarcoma (KS) is not rare in Italy but it is unevenly distributed (Geddes et al, 1994). This pattern seems to be highly dependent on local rates of infection with the not ubiquitous human herpesvirus 8 (HHV-8), which is central in the pathogenesis of KS (Calabrò et al, 1998; Whitby et al, 1998). The reasons underlying geographical variations in HHV-8 sero-prevalence and clustering of classic KS are unclear.

Following the report of an excess of soft tissue sarcomas in the city of Mantua (Costani et al, 2000), an ad hoc survey of all incident cases within the province disclosed a high number of KS cases. An epidemiological study of the disease in this area was therefore carried out. The Mantua province is of special interest because, though not served by a population-based Cancer Registry, it is located in the Po Valley, which has recently been shown to be among the 'hot spots' for HHV-8 infection in Italy (Calabrò et al, 1998; Whitby et al, 1998; Whitby et al, 2000).

### MATERIALS AND METHODS

The province of Mantua (2339 km$^2$, one-eighth of the Po Valley, population: 365,000) in Lombardy (Figure 1) comprises 70 municipalities in 9 administrative 'zones'. It is a flat area of rivers, lakes, marshes and irrigation channels. Mantua's economy is chiefly concerned with the processing of cereal grains (wheat, corn and rice) and livestock farming.

Cases were individuals with a histological diagnosis of KS in the period 1989–1998 (M9140/3, ICD-O morphology code); HIV-related cases were excluded. Incidence rates were computed for each municipality and zone. Direct standardization of incidence rates was performed using the STATA package. Confidence intervals were computed using the Poisson distribution.

The following characteristics were estimated for each municipality: population by age and sex; altitude; urban–rural gradient (a 4-category socio-economic index calculated by applying factor analysis to a subset of 13 variables, including population density and proportion of active population employed in agriculture (ISTAT, 1986) ); main rivers flowing through the territory; whether (or not) it was included among the endemic areas for malaria both in 1902–1903 (Biancorosso, 1935) and 1908 (Soliani, 1909). Cases were coded accordingly to residence at birth, during the first 15 years of life, and main residence. The 3 different incidence rates so computed for the 70 municipalities were treated as outcome variables in a multiple linear regression model, in which the previously mentioned characteristics were present as predictive variables.

### RESULTS

There were classic KS cases in 44 men and 20 women (mean age: 72.2 (range: 26–92), male-to-female ratio: 2.6), as shown in Table 1. The overall standardized incidence rate was 2.5/100 000 in males and 0.7/100 000 in females. Incidence rates showed geographic differences. In the southwest (Viadana/Sabbioneta, zone 9) these were particularly high, both in women (2.8/100 000, 95% CI 2.04–7.84, based on 10 cases) and men (5.0/100 000, 95% CI 2.54–9.10, based on 11 cases). In Table 2, the estimated rates are compared with those of 5 other areas of the Po Valley (Figure 1).

As expected, rates increase with age and appear substantially higher among males though in the oldest age group (≥90), cases were observed only among females, perhaps reflecting the higher mean ages of women.

The multivariate analysis showed that the best-fit model included the independent variables listed in Table 3. Incidence according to residence during the first 15 years of life was the best outcome variable. Zones 4 and 9, which were selected by the model as being significantly associated with the incidence of KS, include 13 municipalities; of these, 9 were formerly endemic for malaria (69%). The corresponding proportion in the other zones is 39% ($P = 0.045$).

### DISCUSSION

Elevated rates of classic KS were found far above those reported for Italy as a whole and for most European countries (Geddes et al, 1994; Zanetti et al, 1997). The estimated rates vary greatly among

*Received 14 February 2001*
*Revised 12 April 2001*
*Accepted 18 April 2001*

*Correspondence to:* V Ascoli

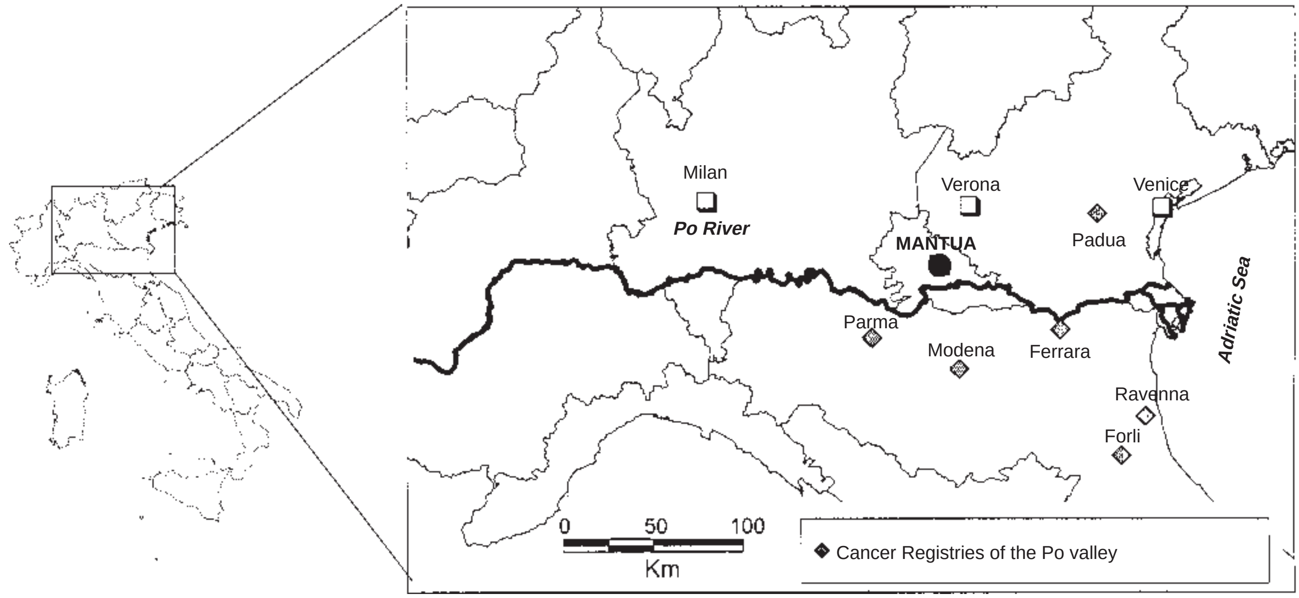

**Figure 1** Location of the province of Mantua (Lombardy), upper Po Valley, Italy

**Table 1** Number of cases[a] and age/sex specific incidence rates of classic Kaposi's sarcoma (per 100 000 person/years) in the Mantua province, Po Valley, Northern Italy, between 1989 and 1998

| | Men | | | Women | | |
|---|---|---|---|---|---|---|
| Age (years) | No. of cases | Population (person/years) | Rate | No. of cases | Population (person/years) | Rate |
| 0–29 | 1 | 644 270 | 0.16 | – | 614 500 | 0.00 |
| 30–39 | 1 | 258 910 | 0.39 | – | 253 790 | 0.00 |
| 40–49 | – | 249 220 | 0.00 | 1 | 247 420 | 0.40 |
| 50–59 | 4 | 239 770 | 1.67 | 1 | 250 120 | 0.40 |
| 60–69 | 12 | 213 300 | 5.63 | 2 | 255 340 | 0.78 |
| 70–79 | 16 | 120 200 | 13.31 | 6 | 181 120 | 3.31 |
| 80–89 | 10 | 41 890 | 23.87 | 8 | 86 710 | 9.23 |
| >90 | – | 1 960 | 0.00 | 2 | 5 860 | 34.13 |
| All | 44 | 1 769 520 | 2.49 | 20 | 1 894 860 | 1.06 |

[a]Only 1 subject was born outside the Po Valley (Southern Italy).

**Table 2** Annual incidence rates[a] of Kaposi's sarcoma (× 100 000 person/years) in the Mantua province and 5 areas of the Po Valley served by a Cancer Registry

| Geographic area | | Kaposi's sarcoma | | | | | |
|---|---|---|---|---|---|---|---|
| | | Males | | | Females | | |
| | | Cases | Rate[d] | Histologic diagnosis (%) | Cases | Rate[d] | Histologic diagnosis (%) |
| Mantua[b] | 1989–98 | 42 | 6.7 | 100 | 19 | 2.6 | 100 |
| Ferrara[c] | 1991–92 | 12 | 8.4 | 100 | 6 | 5.1 | 100 |
| Forli'-Ravenna[c] | 1988–92 | 13 | 3.0 | 95.7 | 8 | 1.5 | 91.7 |
| Modena[c] | 1988–92 | 17 | 3.3 | 84.6 | 6 | 0.9 | 100 |
| Padova[c] | 1988–92 | 18 | 2.6 | 97.1 | 11 | 1.0 | 100 |
| Parma[c] | 1988–92 | 8 | 2.1 | 90.2 | 0 | – | – |

[a]Age-standardized to Italian population of 1991. [b]Not served by a Cancer Registry; HIV-KS excluded. [c]HIV-KS included. [d]Rates are calculated according to the data derived from local Cancer Registries that include HIV-related KS cases (Zanetti et al, 1997). In order to make the figures comparable, age standardized rates are computed considering only cases and populations aged≥50, under the assumption that HIV-related KS cases should be below the age of 50.

 © 2001 Cancer Research Campaign

**Table 3** Multivariate linear regression analysis of incidence rates according to residence during the first 15 years of life. $F_{(6,63)} = 8.77$; $P_F < 10^{-4}$; Adjusted $R^2 = 0.40$

| Variable* | βvalue | $P_t^{§}$ | 95%Confidence interval |
|---|---|---|---|
| 1 river[a] | 0.113 | 0.771 | –0.66–0.88 |
| 2 or more rivers[a] | 0.936 | 0.007 | 0.27–1.61 |
| Semi-urban feature[b] | 0.485 | 0.256 | –0.36–1.33 |
| Semi-rural feature[b] | 1.946 | 0.025 | 0.25–3.64 |
| Rural feature[b] | 1.079 | 0.007 | 0.31–1.85 |
| Zone[c] | 1.865 | $<10^{-3}$ | 1.12–2.61 |
| Constant | –0.343 | 0.377 | –1.11–0.43 |

§*P* value associated with the t value obtained by testing β. [a]Absence of river taken as reference. [b]Urban feature taken as reference. [c]Zones 9 and 4 joined vs. all the others. *Based on municipality of residents.

nearby municipalities, and are particularly high both in men and women in a rural area lying within the confluence triangle of the Oglio and Po rivers (Figure 2). This may reflect geographical variations in the prevalence of HHV-8 infection, but may also be dependent on genetic homogeneity and/or as-yet-unknown environmental agents.

None of the variables considered in the multivariate analysis appears to have a major role in predicting the incidence within the province, except 'belonging to certain geographic areas' where the proportion of municipalities formerly endemic for malaria is significantly higher than in others. The presence of 2 or more rivers and the rural/semi-rural environment may also be relevant.

No data are available regarding HHV-8 seroprevalence in Mantua though several of the co-factors claimed to be associated with an increase in the risk of KS are present such as (i) areas formerly endemic for malaria; (ii) rural lifestyle and cereal farming (Cottoni et al, 1997); (iii) presence of iron oxide-rich clay in soils (Ziegler, 1993); (iv) plenty of blood-sucking insects (McHardy et al, 1984).

There is some evidence that certain zones in the Po Valley are 'endemic' for HHV-8 infection and KS. Incidence rates in Ferrara (delta of the Po River) are among the highest in the world (Zanetti et al, 1997). Classic KS has been documented among Po Valley people from Lombardy (Rabbiosi, 1959; Zanca et al, 1973; Brambilla et al, 1988), Emilia-Romagna (Martinotti, 1938) and Piedmont (Vineis et al, 1987). More recently, serological (Calabrò et al, 1998; Whitby et al, 1998; Whitby et al, 2000) and molecular-based studies (Luppi et al, 1996; Monini et al, 1996) have shown that HHV-8 infection is present in healthy subjects of the Po Valley. Our findings contribute to the mapping of classic KS in Italy and, besides the south of Italy, Sardinia and Sicily, indirectly confirm the Po Valley as a high-risk area. A sero-epidemiological survey of the prevalence of HHV-8 infection in the KS-free population is in progress.

### ACKNOWLEDGEMENTS

The authors wish to thank Professor Mario Coluzzi (La Sapienza University, Rome) for his contribution on data concerning malaria and Mrs Luciana Gatti (Local Health Unit, Mantua) for her co-operation in data collection.

### REFERENCES

Biancorosso R (1935) *La legislazione italiana sulla malaria*. S. A. Bevilacqua-Lombardini: Forli

Brambilla L, Boneschi V, De Blasio A, Chiappino G, Melotti E and Hepeisen S (1988) Mediterranean Kaposi's sarcoma. Associated pathology in a case study of 100 patients. *G Ital Dermatol Venereol* **123**: 477–480

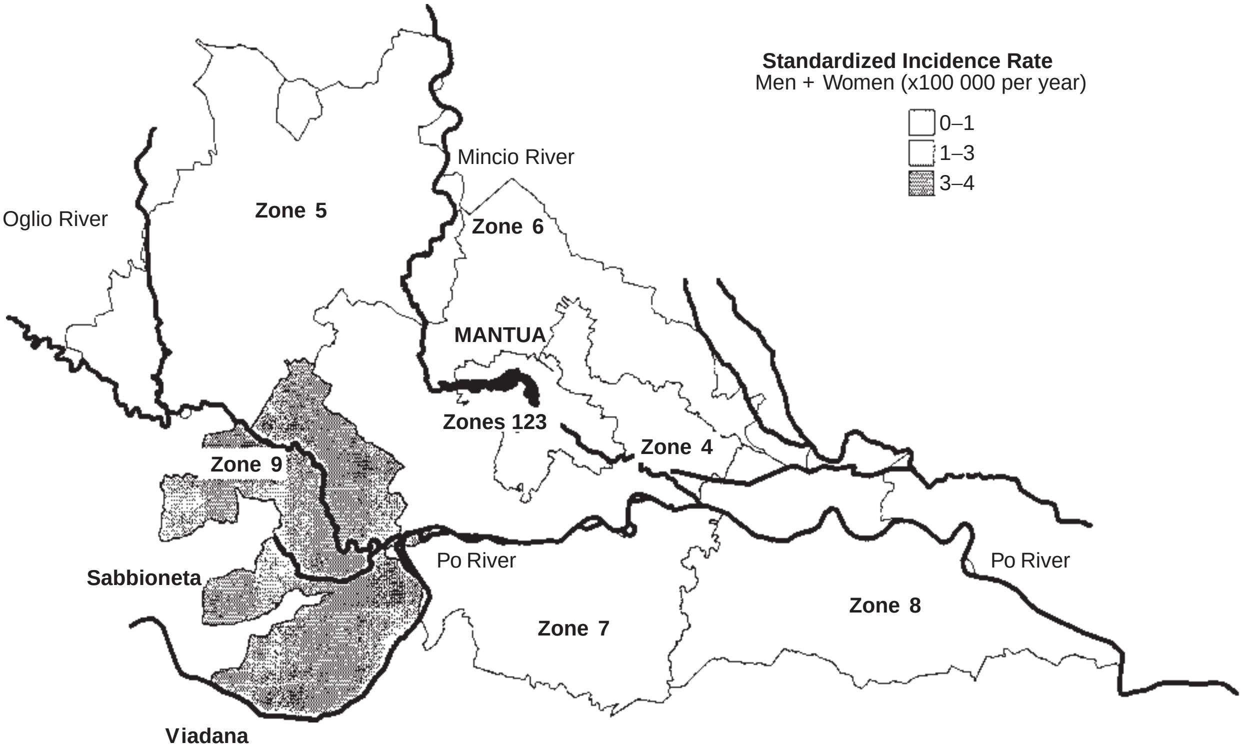

**Figure 2** Geographical distribution of standardized incidence rates by zone, for men and women together

© 2001 Cancer Research Campaign

Calabrò ML, Sheldon J, Favero A, Simpson GR, Fiore JR, Gomes E, Angarano G, Chieco-Bianchi L and Schulz TF (1998) Seroprevalence of Kaposi's sarcoma-associated herpesvirus/human herpesvirus 8 in several regions of Italy. *J Hum Virol* **1**: 207–213

Costani G, Rabitti P, Mambrini A, Bai E and Berrino F (2000) Soft tissue sarcomas in the general population living near a chemical plant in northern Italy. *Tumori* 2000 **86**: 381–383

Cottoni F, De Marco R and Montesu MA (1996) Classical Kaposi's sarcoma in north-east Sardinia: an overview from 1977 to 1991. *Br J Cancer* **72**: 1132–1133

Cottoni F, Masala MV, Budroni M, Rosella M, Satta R, Locatelli F, Montesu MA and De Marco R (1997) The role of occupation and a past history of malaria in the etiology of classic Kaposi's sarcoma: a case-control study in north-east Sardinia. *Br J Cancer* **76**: 1518–1520

Geddes M, Franceschi S, Barchielli A, Falcini F, Carli S, Cocconi G, Conti E, Crosignani P, Gafà L, Giarelli L, Vercelli M and Zanetti R (1994) Kaposi's sarcoma in Italy before and after the AIDS epidemic. *Br J Cancer* **69**: 333–336

Geddes M, Franceschi S, Balzi D, Arniani S, Gafà L and Zanetti R (1995) Birthplace and classic Kaposi's sarcoma in Italy. *J Natl Cancer Inst* **87**: 1015–1017

ISTAT (1986) *Classificazione dei comuni secondo le caratteristiche urbano rurali. Note e Relazioni*. ISTAT: Roma

Luppi M, Barozzi P, Maiorana A, Collina G, Ferrari MG, Marasca R, Morselli M, Rossi E, Ceccherini-Nelli L and Torelli G (1996) Frequency and distribution of herpesvirus-like DNA sequences (KSHV) in different stages of classic Kaposi's sarcoma and in normal tissues from an Italian population. *Int J Cancer* **66**: 427–431

Martinotti L (1938) Sarcoma di Kaposi famigliare. In *Archivio Italiano di Dermatologia, Sifilografia e Venerologia*. XIV: 367–369. Cappelli L: Bologna

McHardy J, Williams EH, Geser A, de-The G, Beth E and Giraldo G (1984) Endemic Kaposi's sarcoma: incidence and risk factors in the West Nile District of Uganda. *Int J Cancer* **15**: 203–212

Monini P, de Lellis L, Fabris M, Rigolin F and Cassai E (1996) Kaposi's sarcoma-associated herpesvirus DNA sequences in prostate tissue and human semen. *N Engl J Med* **334**: 1168–1172

Rabbiosi G (1959) Considerazioni su alcuni casi familiari di angioreticulosi cutanea (sarcomatosi multipla idiopatica di Kaposi). *Dermatol* **10**: 185–186

Soliani G (1909) La malaria in città e provincia di Mantova negli anni 1907 e 1908. In *Atti della Società per gli Studi della Malaria* X: pp 383–392

Vineis P, Terracini B, Ciccone G, Cignetti A, Colombo E, Donna A, Maffi L, Pisa R, Ricci P, Zanini E and Comba P (1987) Phenoxy herbicides and soft-tissue sarcomas in female rice weeders. A population-based case-referent study. *Scand J Work Environ Health* **13**: 9–17

Whitby D, Luppi M, Barozzi P, Boshoff P, Weiss RA and Torelli G (1998) Human herpesvirus-8 seroprevalence in blood donors and lymphoma patients from different regions of Italy. *J Natl Cancer Inst* **90**: 395–397

Whitby D, Luppi M, Sabin C, Barozzi P, Di Biase AR, Balli F, Cucci F, Weiss RA, Boshoff C and Torelli G (2000) Detection of antibodies to human herpesvirus 8 in Italian children: evidence for horizontal transmission. *Br J Cancer* **82**: 702–704

Zanca A and Giubertoni G (1973) Su un caso di associazione della malattia di Kaposi con la leucemia linfatica cronica. *Giorn It Derm* **108**: 542–556

Zanetti R, Crosignani S and Rosso S (1997) *Il cancro in Italia. I dati di incidenza dei Registri Tumori, 1988–1992*. Il Pensiero Scientifico: Roma

Ziegler JL (1993) Endemic Kaposi's sarcoma in Africa and local volcanic soils. *Lancet* **342**: 1348–1351

 *© 2001 Cancer Research Campaign*

